# Exploiting the Physicochemical and Antimicrobial Properties of PHB/PEG and PHB/PEG/ALG-e Blends Loaded with Ag Nanoparticles

**DOI:** 10.3390/ma15217544

**Published:** 2022-10-27

**Authors:** Mário R. P. da Silva, Robert S. Matos, Michael D. S. Monteiro, Samuel B. Santos, Henrique D. F. Filho, George R. S. Andrade, Marco Salerno, Luís E. Almeida

**Affiliations:** 1 Postgraduate Program in Materials Science and Engineering, Federal University of Sergipe-UFS, São Cristovão 49100-000, Sergipe, Brazil; 2 Laboratory of Corrosion and Nanotechnology (LCNT), Federal University of Sergipe, São Cristovão 49100-000, Sergipe, Brazil; 3 Postgraduate Program in Physiological Sciences, Federal University of Sergipe-UFS, São Cristovão 49100-000, Sergipe, Brazil; 4 Laboratory of Synthesis of Nanomaterials and Nanoscopy (LSNN), Federal University of Amazonas-UFAM, Manaus 69077-000, Amazonas, Brazil; 5 Postgraduate Program in Energy, Federal University of Espírito Santo, São Mateus 29075-910, Espírito Santo, Brazil; 6 Institute for Globally Distributed Open Research and Education (IGDORE), Institute for Materials Science, Max Bergmann Center of Biomaterials, Technische Universität Dresden, 01069 Dresden, Germany

**Keywords:** poly(3-hydroxybutyrate), polyethylene glycol, esterified alginate, nanocomposites, antibacterial activity

## Abstract

Poly(3-hydroxybutyrate) (PHB)-based films containing Poly(ethylene glycol) (PEG), esterified sodium alginate (ALG-e) and polymeric additives loaded with Ag nanoparticles (AgNPs) were obtained by a conventional casting method. AgNPs were produced in aqueous suspension and added to polymeric gels using a phase exchange technique. Composite formation was confirmed by finding the Ag peak in the XRD pattern of PHB. The morphological analysis showed that the inclusion of PEG polymer caused the occurrence of pores over the film surface, which were overshadowed by the addition of ALG-e polymer. The PHB functional groups were dominating the FTIR spectrum, whose bands associated with the crystalline and amorphous regions increased after the addition of PEG and ALG-e polymers. Thermal analysis of the films revealed a decrease in the degradation temperature of PHB containing PEG/AgNPs and PEG/ALG-e/AgNPs, suggesting a catalytic effect. The PHB/PEG/ALG-e/AgNPs film combined the best properties of water vapor permeability and hydrophilicity of the different polymers used. All samples showed good antimicrobial activity in vitro, with the greater inhibitory halo observed for the PEG/PEG/AgNPs against Gram positive *S. aureus* microorganisms. Thus, the PHB/PEG/ALG-e/AgNPs composite demonstrated here is a promising candidate for skin wound healing treatment.

## 1. Introduction

Over the last few decades, investigations regarding skin wounds caused or aggravated by bacterial infections of different natures have been increased due to the long-term resistance of microorganisms to antibiotics [1]. Without a suitable treatment, skin wounds can evolve rapidly and in some cases, such as diabetes disease, they can lead to amputation or death of the patient [2,3]. The use of traditional dressings, e.g., alginate, hydrogels, and hydrocolloids, as a complementary strategy to antibiotics has been shown to be ineffective because the dressings are not able to reduce bacterial burden [4]. Thus, the increase in the resistance of bacteria to antibiotics and the ineffectiveness of traditional dressings are current problems that hamper the treatment of bacterial infection-induced chronic skin wounds.

Recently, the tissue engineering field has been dedicated to the development of biocompatible antimicrobial dressings [5,6]. For this purpose, several biopolymers have been used as the main biocompatible matrix, considering their physicochemical (e.g., porosity, water vapor permeability, wettability, elasticity, etc.) and antimicrobial properties [7,8]. In this regard, poly(3-hydroxybutyrate) (PHB) is an interesting biodegradable and biocompatible biopolymer synthesized by bacterial fermentation, but its crystalline properties hinder its promising rheological behavior as a biomaterial [9]. The use of PHB as a biocompatible material can be improved by induction of amorphization of its structure with the introduction of other biodegradable polymers [4,10]. Among them, poly(ethylene glycol) (PEG) and sodium alginate (ALG) are hydrophilic and amorphous biodegradable biopolymers widely used as additives for the fabrication of blends with improved mechanical properties for biomedical purposes [8,11,12,13]. PEG is a known synthetic plasticizer that displays low toxicity, which has motivated its application for drug controlled release and tissue regeneration [14,15,16]. ALG, in turn, is a highly hydrophilic biopolymer, which can be a problem for the production of blends with PBH due to their low compatibility, as PHB is a more hydrophobic biopolymer [17]. Despite that, ALG can be esterified (to ALG-e) with alcohols of different chains, which confers an amphiphilic character that can enable a better homogenization with PHB [8]. However, so far, there are very few studies based on the fabrication and physicochemical characterization of blends with these three biopolymers.

Notably, the development of antifouling and active self-disinfecting surfaces has arisen as an alternative and sustainable promising approach that can be used in modern biomedicine [18]. The literature has reported that several functional polymers have an intrinsic condition of acting as an antibacterial agent, generating active surfaces that hinder bacterial adhesion and promote the release of bacterial debris [19]. However, the use of most biopolymers without loading a known antimicrobial agent does not guarantee that the blends exhibit efficient antimicrobial activity [20]. For this reason, in recent investigations, Ag nanoparticles (AgNPs) [21,22] or Ag ions [23] have been incorporated into blend structures in order to improve their antimicrobial properties as a whole. The rough hydrophobic PVA/PVP/Ag nanocomposites fabricated by El-Kader et al. [24] produced zones of inhibition ranging from 13–17 mm against both *S. aureus* and *E. coli* microorganisms. The chitosan/alginate blends loaded with AgNPs obtained by Chabala et al. [25] were able to produce zones of inhibition on their films against *E. coli* and *S. aureus* similar to antibiotics such as tetracycline and gentamicin. Alipour et al. [26] synthesized PVA/PVP/Pectin/Mafenide blends loaded with AgNPs that were considered as responsible for the appearance of zones of inhibition that ranged from 2.5–5 mm against *E. coli* and *P. aeruginosa* strains. These previous works prove that the incorporation of AgNPs can lead to an improvement in the antimicrobial properties of blends of interest for the regenerative medicine field.

Herein, we have fabricated PHB films containing PEG and ALG-e additives loaded with AgNPs by conventional casting. Our main goal was to investigate the physicochemical and antimicrobial properties of the composites. The films were characterized by several experimental techniques, and antimicrobial tests were performed using the agar diffusion method. The results revealed that the prepared formulation has promising physicochemical properties and antimicrobial activity.

## 2. Materials and Methods

### 2.1. Materials

PHB (molar weight 528.265 g∙mol^−1^, poly dispersion index 2, hydroxyvalerate content 4%, purity ≥ 99.57%, and density 1.23 g∙cm^−3^) was purchased from PHB Industrial S/A, São Paulo, Brasil, to be used as the main polymer of the films. Furthermore, chloroform (Panreac, purity ≥ 99.98%), n-butyl alcohol (Neon, purity ≥ 99.95%), sodium alginate (ALG) (Dinâmica, purity ≥ 99%), PEG1500 (Synth, PA), sodium borohydride (Vetec, purity ≥ 98%), sodium citrate (Sigma-Aldrich, purity ≥ 99%), and silver nitrate (Sigma-Aldrich, purity ≥ 99.5%) were also used for film synthesis.

### 2.2. Synthesis of AgNPs

The AgNPs were synthesized using a previously reported method [27], but with modifications. Firstly, 100 mL of a sodium citrate (0.31 mM) solution was mixed with 100 mL of a silver nitrate solution and kept under vigorous stirring. After that, 6 mL of a sodium borohydride (0.25 mM) solution was added into the resultant solution, which was slowly stirred for 10 min. The final precursor solution was gradually heated to boiling in a liquid silicone bath for 90 min, resulting in a suspension of AgNPs.

### 2.3. Sodium Alginate Esterification

The esterification of ALG was performed according to a previously reported method [8]. In a typical procedure, 5 g of ALG was dissolved in 185 mL of n-butanol alcohol. Afterward, 1 mL of H_2_SO_4_ was added, and the resultant solution was kept under stirring at 50 °C for 6 h. The final solution was dried for 3 days at room temperature, resulting in esterified alginate (ALG-e). The ALG-e proportion was determined by the Broderick et al. [28] protocol. In summary, 0.5 g of dried ALG-e was dissolved in 50 mL of a solution composed by ultrapure water and ethanol (25:75 %v). The solution was homogenized in a water bath under stirring at 50 °C for 30 min. Subsequently, 40 mL of NaOH (0.5 mol∙L^−1^) solution was added to the solution, which was maintained under stirring at 100 °C for 15 min. The resulting solution was cooled to room temperature and titrated using a HCl (0.5 mol∙L^−1^) solution. The ALG-e esterification degree (ED) (%) was determined as follows [28]:

(1) $$\mathrm{ED}\left(\%\right)=\frac{\left(V_{o}-V_{p}\right)\times {\;M}_{\mathrm{HCl}}\times 73}{m}\times 100\;$$

where V_o_ is the volume of HCl used in the titration of the NaOH solution, V_p_ is the volume of HCl used in the titration of the saponified polymer sample, MHCl is the molarity of the HCl, 73 is the molar mass of the butyl ion, and m is the mass of the sample.

### 2.4. Synthesis of the Films

The film-forming gels were obtained according to a previously reported method [8]. In summary, the polymeric gels were prepared using known masses of PHB, PEG, and ALG-e in 50 mL of chloroform, whose relative amounts are reported in Table 1. The homogenization of the mixtures was performed under stirring and heating at 80 °C for 24 h. The phase exchange method reported by Manivela and Anandan [29] was used for loading AgNPs into the precursor gel structure. In a typical procedure, each film-forming gel was mixed with the AgNPs suspension at a ratio 1:1 (*v*/*v*) and kept under vigorous stirring for 24 h. The final gels loaded with AgNPs were separated using a decanting funnel for 24 h. Afterward, the casting technique was applied to the fabrication of PHB/AgNPs, PHB/PEG/AgNPs, and PHB/PEG/ALG-e/AgNPs composites.

### 2.5. Characterization of the AgNPs

The absorption spectra of the samples in aqueous suspension were obtained using a Perkin Elmer Lambda 45 spectrophotometer, operating in the wavelength rage of 200–800 nm. The percentage of AgNPs adorbed into the gel structure (PAg (%)) was evaluated from UV-vis spectra using the following formula:

(2) $$P_{\mathrm{Ag}}\left(\%\right)=\frac{A_{i}-A_{f}}{A_{i}}\times 100$$

where $A_{i}$ and $A_{f}$ are the absorbance of the suspension before and after the phase exchange procedure. 

The morphological characterization of the AgNPs in solution was carried out using a solution cast on Cu grids in a transmission electron microscope (TEM) by using a Joel JEM1400plus instrument (Joel solutions for innovation, Peabody, MA, USA), operating at 120 kV.

### 2.6. Characterization of the Composite

The functional groups present in ALG-e and composites were determined by Fourier transform infrared spectroscopy (FTIR) in a Nicolet (iS-10) equipment operating in ATR mode in the wavenumber range of 2000–700 cm^−1^, with a resolution of 4 cm^−1^. 

The X-ray diffraction (XRD) patterns of the films were obtained in a Shimadzu (LabX XRD-6000) diffractometer, operating with CuKα radiation (λ = 1.54056 Å), a nickel filter with voltage of 40 kV, current of 40 mA, scanning speed of 2°∙min^−1^, and a scattering angle 2θ = 10–35°. 

The morphology of the films was evaluated by scanning electron microscopy (SEM) in a Jeol-JSM 6390 (Jeol, MA, USA) apparatus. 

Thermogravimetric analysis was carried out on a TGA/DTA simultaneous thermal analyzer (NETZSCH STA 449 F1 Jupiter) in a nitrogen atmosphere with a flow of 100 mL∙min^−1^, operating at 25–550 °C with a heating rate of 10 °C∙min^−1^.

The water vapor permeation (WVP) of the films was evaluated based on the international standard ASTM E 96/E96 M-05 [30] described by Ning et al. [31], but with modifications. The films were placed in a sealed recipient containing originally 3.5 ± 0.1 g of calcium chloride. After that, the films were stored in a desiccator containing a saturated sodium chloride solution. The calcium chloride mass was weighed every 24 h for a total of 7 days. To determine the water vapor transmission rate (WVPR) we used the following formula:

(3) $$\mathrm{WVPR}=\frac{\Delta m}{\Delta t\;\times \;A}$$

where A is the area of the film exposed, Δm is the mass variation, and Δt is time variation. After a period of 7 days, the following formula was used to determine the WVP of the films:

(4) $$\mathrm{WVP}=\frac{\mathrm{WVPR}}{S\left(R1-R2\right)}\times T$$

where S is the water vapor saturation pressure (Pa) at the temperature at which the test was performed, R_1_ is the relative humidity of the desiccator, R_2_ is the relative humidity inside the sealed recipient, and T is the film thickness. Our experiments were performed in triplicate (N = 3) to obtain the representative average and standard deviation values of WVP and WVPR.

The contact angle of the composite films was obtained using a sessile drop method, using a digital camera (Haiz, China) with a focus range of 0–200 mm. The drop images were taken at room temperature and relative humidity of 50 ± 3%. Typically, four measurements (N = 4) were carried out on random regions of the films surface to obtain the representative average and standard deviation values of the contact angles. The obtained images were analyzed using ImageJ version 1.53k freeware software (Java version 1.8.0_172) (www.imagej.nih.gov/ij/, accessed on 27 May 2022).

### 2.7. Antimicrobial Essays

Microbial agar diffusion (Mueller–Hinton CLSI, 2005) susceptibility tests were performed to evaluate the antimicrobial activity of the films against standard strains of *Staphylococcus aureus* ATCC 25923 and *Escherichia coli* (ATCC 25922). Our protocol was based on the procedures previously reported by Bauer et al. [32]. In summary, overnight cultures (35 ± 2 °C) in 5 mL Trypticase^®^ Soy Broth (TSB) were standardized in the scale of 0.5 Mac Farland. Approximately 100 µL of each culture were inoculated into Petri dishes containing 4 mm of previously solidified Muller–Hinton agar (pH 7.2–7.4) and spread with a sterile Drigalsky’s loop. Subsequently, the discs with 1 cm^2^ of diameter were placed on the surface of the medium containing the microorganisms. The constituents of the films were considered as a negative control (CLSI) [33], while gentamicin (10–120 µg∙mL^−1^) was used as a positive control due to its known antimicrobial activity against ATCC standard strains. The antimicrobial activity of each film was evaluated by the formation of an inhibition halo, whose diameter size was measured using a caliper. Our essays were performed in triplicate (N = 3) to obtain the representative average and standard deviation values of the halo of antimicrobial inhibition.

### 2.8. Statistical Analysis

The results were performed in triplicate and expressed as average value, whose statistical significance was evaluated by one-way analysis of variance (ANOVA) pair-comparisons according to Tukey test with a significance level of 95% (*), 99% (**) and 99.9% (***), using the commercial software Origin© 8.0 SRO OriginLab Corporation (www.originlab.com, accessed on 27 may 2022).

## 3. Results and Discussion

### 3.1. AgNPs Characterization

**Figure 1:**
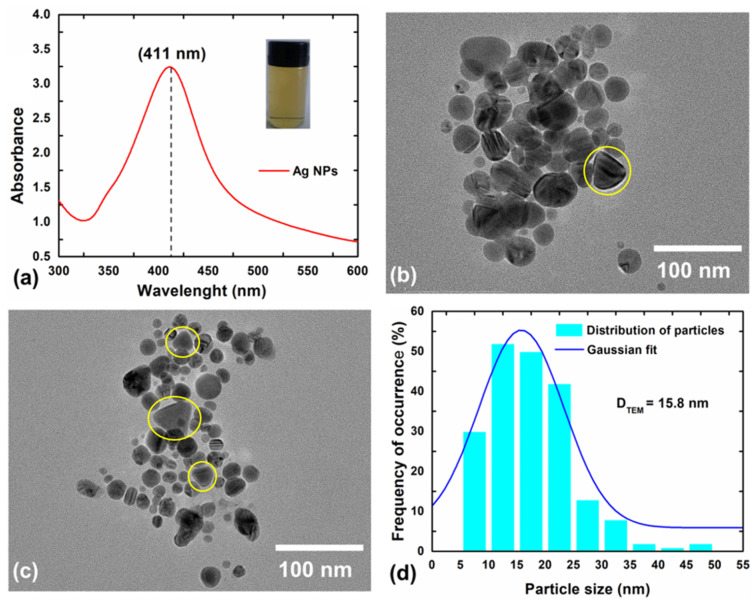
(**a**) UV-vis spectrum, (**b**,**c**) TEM images, and (**d**) Particle size distribution of AgNPs passivated using citrate in the aqueous medium. The yellow circles highlight the anisotropic prismatic shapes observed for some nanoparticles.

The solution resulting from the mixture of sodium citrate, silver nitrate, and sodium borohydride, which was initially colorless, gradually changed until reaching a final yellowish color after ~90 min. The optical properties of this solution and the morphological properties of the resulting NPs were analyzed, and the results are shown in Figure 1. The visual change of color of the AgNPs precursor solution indicates that the conversion from Ag^+^ into Ag^0^ (in nanometer size) was successful, and its appearance is shown in the image inside the UV-vis curve (Figure 1a). As one can see, the UV-vis curve displays a band positioned at 411 nm (~3 eV), which is commonly associated with the plasmon resonance of AgNPs [34,35,36,37,38,39]. Moreover, the sharp aspect of the band suggests the formation of spherical and uniform-sized NPs, with little inhomogeneous broadening [40,41,42].

**Figure 2:**
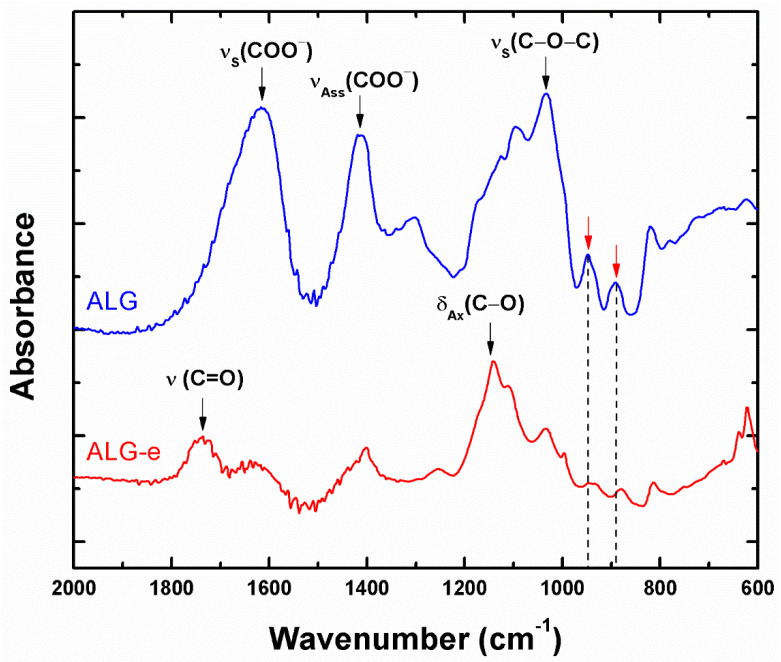
FTIR spectra of alginate (ALG) and esterified alginate (ALG-e).

Indeed, the TEM images (Figure 2) reveal the predominance of spherical-shaped structures. However, it is also possible to observe the presence of NPs with anisotropic morphologies, such as nanoprisms (highlighted by the yellow circles). According to Yu et al. [43], the transformation of spherical to prism-like structures in the Ag nanostructures occurs due to coalescence of smaller spherical NPs facilitated by an excess of reducing agent. The average size of the AgNPs was estimated using a Gaussian fit based on a distribution that considered a total of 200 NPs. The value was found to be ~16 nm, showing that the particles obtained are of nano-scale size, a favorable characteristic for antimicrobial and antibiofilm applications [40,41].

### 3.2. ALG Esterification

Before preparing the PHB/PEG/ALG blend, the ALG was esterified (ALG-e) in order to improve the PHB-ALG interaction. The esterification degree (ED, Equation (1)) was determined by titration, and turned out to be 39.3%, which is a similar result to that reported previously by Lopes et al. [8] (37%). A useful comparison of ALG and ALG-e chemical composition was obtained using FTIR spectra, as shown in Figure 2. Moreover, the result of the comparative analysis is summarized in Table 2. The results show that the esterification process successfully replaced free carboxylic groups with alkyl groups, leaving the ALG structure with a hydrophobic character, but maintaining its hydrogel-forming property and a relative amount of Na^+^ ions in its structure. This is a necessary condition for its application in regenerative medicine [7].

The ALG spectrum displays bands positioned at 944 and 892 cm^−1^ that are due to small amounts of guluronic and mannuronic acids (highlighted by red arrows), respectively. A consequence of the esterification process was the reduction of these bands, suggesting that this process also involved the functional groups of these residues. Furthermore, the bands located at 1033, 1416, and 1614 cm^−1^ are assigned to vibrations of C–O groups of alcohol and symmetrical and asymmetrical deformations of carboxyl groups COO^−^, respectively [8,28]. In the ALG-e spectrum, there is a band at 1735 cm^−1^, which is ascribed to carbonyl (C=O) present in ester [8,28]. Additionally, another band observed at 1138 cm^−1^ can be attributed to the axial deformation of C–O in ester groups [28]. Therefore, these results prove that ALG was successfully converted to ALG-e, with a suitable ED.

### 3.3. Characterization of the Films

#### 3.3.1. Evaluation of AgNPs Loading

**Figure 3:**
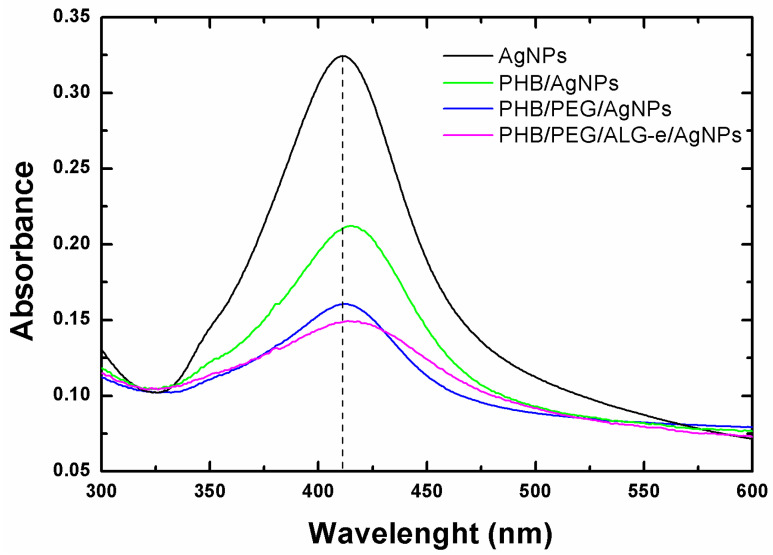
UV-vis spectra of supernatants from each film-forming suspension containing AgNPs.

As previously reported, the phase exchange method was applied to obtain PHB, PHB/PEG, and PHB/PEG/ALG-e film-forming gels loaded with AgNPs. The effective loading of AgNPs in the structure of their gels was evaluated using the UV-vis spectra of the supernatant resulting from the phase exchange process, whose results are displayed in Figure 3. The presence of the characteristic absorption peak characteristic of the AgNPs reveals the effective loading of the AgNPs. Additionally, on loading the AgNPs into the polymer matrix there was only minor red-shift (observed for PHB/AgNPs and especially for the PHB/PEG/ALG-e/AgNPs), limited to maximum ~5 nm, with little intensity decrease and peak broadening (FWHM from 83 nm to 94 nm), suggesting that there was no agglomeration of the NPs. Based on these spectra, the percentage of AgNPs adsorbed in the gels was found to be ~36 (PHB/AgNPs), ~46 (PHB/PEG/AgNPs), and ~54% (PHB/PEG/ALG-e/AgNPs). Therefore, the PHB/PEG/ALG-e matrix has the highest amount of AgNPs in its structure, showing that PEG and ALG-e, as polymeric additives, help the adsorption and stabilization of AgNPs in the gel structure [44,45].

#### 3.3.2. Structural and Morphological Analysis

**Figure 4:**
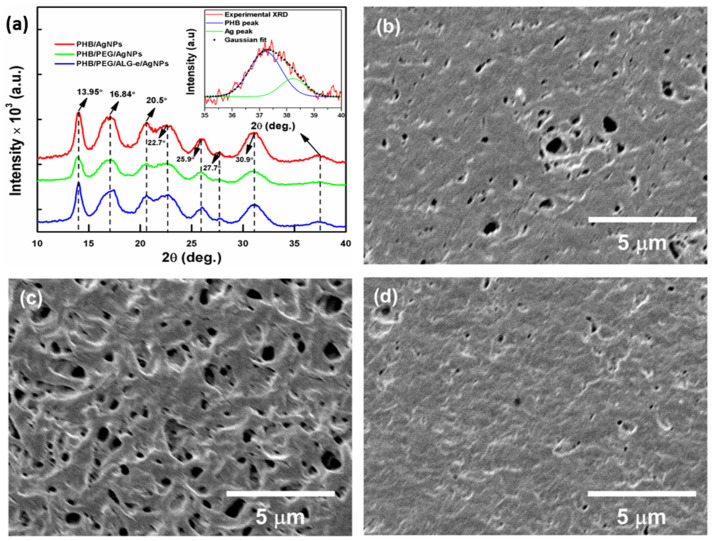
(**a**) XRD patterns and (**b**–**d**) SEM images of PHB/AgNPs, PHB/PEG/AgNPs, and PHB/PEG/ALG-e/AgNPs films, respectively. The plot inside panel (**a**) shows the deconvolution of the region comprising the superposition of PHB and Ag peaks in the range of 35–40°.

The results of the structural and morphological analysis of the films are presented in Figure 4. It is known that PHB crystallizes in an orthorhombic-like structure [46] belonging to the P212121 space group [47]. In the XRD patterns shown in Figure 4a, some of the main reflections associated with the PHB polymer matrix can be found. Explicitly, all spectra display similar patterns, with reflections centered around 2θ angles of 13.95°, 16.84°, 20.5°, 22.7°, 25.9°, 27.7°, and 30.9°, which are due to the crystallographic planes (020), (110), (101), (111), (121), (040), and (002), respectively. Previous works have shown that these planes typically occur for the ordered orthorhombic structure of poly-β-hydroxybutyrate [46,48,49]. The patterns also exhibit a large halo positioned in the range of 35°–40°, which was also observed previously by D’Amico et al. [50]. This makes it difficult to identify reflections associated with the AgNPs. However, the PHB/AgNPs pattern (plot inside Figure 4a) was deconvolved using a Gaussian function and revealed the existence of two peaks positioned at 37.3° and 38.1°, which arise thanks to the coexistence of PHB and AgNPs. The peak positioned around 38.1° is ascribed to the (111) plane of face-centered cubic (FCC) arrangement of Ag [51], confirming that AgNPs are loaded in the polymeric matrix of PHB. Additionally, the PHB/PEG/AgNPs film exhibits a (020) plane peak that is broader than the one for the PHB/AgNPs and PHB/PEG/ALG-e/AgNPs films, suggesting that there is less organization of polymeric chains in this film. Notably, the PHB/PEG/ALG-e/AgNPs film appears to have the narrowest peak, indicating a higher crystallinity of this system.

Regarding the films’ morphology, Figure 4b–d reveal the typical surface of the different PHB films obtained by the casting method, which is in good agreement with the previous report of Lopes et al. [8]. In particular, the surface of the PHB/PEG/AgNPs film (Figure 4c) is covered by irregular pores of different sizes, which are also visible—even though more moderately—for the PHB/AgNPs film (Figure 4b). These surface pores can be associated with defects generated by the casting procedure or voids left during drying of the films, which are caused by removal of the volatile solvent [52]. Conversely, the surface of the PHB/PEG/ALG-e/AgNPs film (Figure 4d) shows almost no pores, suggesting that the presence of ALG-e provided a reduction in their number. From a physicochemical point of view, a slower kinetics of solvent evaporation in the films containing ALG-e can be the reason for the reduction of the surface pores. In contrast, the presence of AgNPs in the structure of the films does not influence their surface morphology, as no significant morphological changes can be observed in these different samples.

#### 3.3.3. FTIR and TGA/DTG Analysis

**Figure 5:**
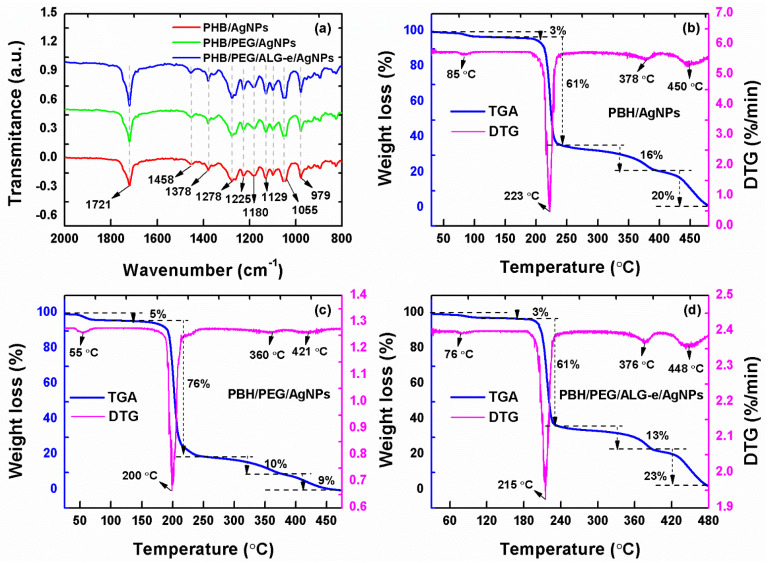
(**a**) FTIR spectra and (**b**–**d**) TGA/DTG curves of PHB/AgNPs, PHB/PEG/AgNPs, and PHB/PEG/ALG-e/AgNPs films.

The FTIR spectra of the films are shown in Figure 5a, which reveals the persistent presence of several common functional groups in the blends, whose band positions are summarized in Table 3. The band centered at 1721 cm^−1^ corresponds to the axial deformation of the C=O ester group [53]. At 1278 and 1129 cm^−1^ there are bands assigned to the axial deformation of the C–O–C ester group [54]. The stretching vibration of the C–O group located at 1055 cm^−1^ and the bands between 1458 and 1378 cm^−1^ due to the asymmetric and symmetric angular deformations of –CH_3_ groups [55,56] are also ascribed to the PHB polymer. Furthermore, the band positioned at 979 cm^−1^ is associated with the axial deformation of the C–C bond [56] due to the PHB. Notably, both bands of amorphous (1278 cm^−1^) and crystalline (1721 cm^−1^) PHB increase after the addition of ALG-e. The increase in crystallinity in the crystalline regions could be due to chelation of AgNPs in the PHB matrix, and the increase in amorphism can be associated with the incorporation of the amorphous matrices of PEG and ALG-e.

The thermal analysis of each film is shown in Figure 5b–d. The films exhibit a total of four well-defined thermal mass loss events, and above 450 °C they are completely degraded. The initial weight loss ranging from 3–5% below 100 °C in all films can be attributed to the release of adsorbed water [58]. The most pronounced weight loss occurred between 200 and 250 °C, where a total loss of 61, 76, and 61% can be observed for the PHB/AgNPs, PHB/PEG/AgNPs, and PHB/PEG/ALG-e/AgNPs films, respectively. It is known that the thermal decomposition of PHB occurs between 250 and 300 °C, forming degradation products consisting of crotonic acid and low molecular weight oligomers [8]. In this regard, PHB degradation occurs by random cleavage in the ester bond at temperatures above 200 °C, resulting in a decrease in polymeric molar mass [59,60,61]. Moreover, the films exhibit onset temperature (T_onset_) at 223 °C, 200 °C, and 215 °C for PHB/AgNPs (Figure 5b), PHB/PEG/AgNPs (Figure 5c), and PHB/PEG/ALG-e/AgNPs (Figure 5d), respectively. This reveals that there is a decrease in the thermal stability of the blends after loading with Ag-NPs. The chain-scission of the PHB polymer is also observed by additional stages of its degradation between 360 and 450 °C that lead to a final weight loss of 36% (PHB/AgNPs), 19% (PHB/PEG/AgNPs), and 36% (PHB/PEG/ALG-e/AgNPs). The comparative analysis shown in Table 4 also reveals that the T_onset_ of all AgNPs-loaded films are significantly smaller than for unloaded films produced by Lopes et al. [8]. This significant decrease in the T_onset_ of the films can be ascribed to the presence of AgNPs, which decrease the activation energy of the polymeric chains [62] and promote a catalytic effect [63] that further accelerates the films degradation. Despite that, the polymeric composition of the blends also dictates their thermal behavior, which is in complete agreement with the observations of Lopes et al. [8], which showed that PHB/PEG and PHB/PEG/ALG-e blends have lower T_onset_ compared to pure PHB polymer. In fact, the low T_onset_ of PEG (257 °C) [59] and ALG-e (220 °C) [60,61,64] compared to pure PHB polymer (281 °C) [8] may also be behind the unique thermal behavior of PHB-based blends loaded with AgNPs. Thus, the coexistence of PEG, ALG-e, and AgNPs in the PHB matrix plays an important role in reducing the thermal stability of the films.

#### 3.3.4. Water Vapor Permeability

**Figure 6:**
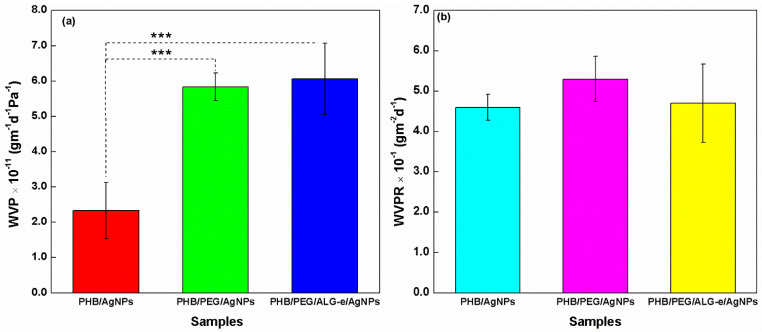
(**a**) Water vapor permeability (WVP) and (**b**) water vapor permeability rate (WVPR) of PHB/AgNPs, PHB/PEG/AgNPs, and PHB/PEG/ALG-e/AgNPs films. *** means that there was statistical difference between the samples with significance level *p* < 0.001.

The WVP and WVPR of the films are shown in Figure 6. The PHB/AgNPs film exhibited the lowest WVP (2.3 × 10^−11^ ± 0.8 × 10^−11^ gm^−1^d^−1^Pa^−1^), while the PHB/PEG/AgNPs and PHB/PEG/ALG-e/AgNPs films showed the highest values of 5.8 × 10^−11^ ± 0.4 × 10^−11^ gm^−1^d^−1^Pa^−1^ and 6.1 × 10^−11^ ± 0.1 × 10^−11^ gm^−1^d^−1^Pa^−1^ after 7 days, respectively (Figure 6a). However, the average values obtained by the PHB/PEG/AgNPs and PHB/PEG/ALG-e/AgNPs films showed no significant difference (*p* > 0.05), proving that these films have the same characteristics of water vapor permeation, but they are different compared to PHB/AgNPs films (*p* < 0.05). Additionally, all films have similar WVPR, as no significant difference was observed between the average values obtained (Figure 6b) (*p* > 0.05) after 24 h, although the PHB/PEG/AgNPs film presents a more porous structure, as shown in the SEM analysis (Figure 4c). Thus, the addition of plasticizing PEG and ALG-e compounds causes greater water vapor permeability in the film structure, but with similar transmission rates of 46 ± 3 gm^−2^d^−1^ (PHB/AgNPs), 53 ± 6 gm^−2^d^−1^ (PHB/PEG/AgNPs) and 47 ± 10 gm^−2^d^−1^ (PHB/PEG/ALG-e/AgNPs). Our analysis is in full agreement with Lopes et al. [8], whose PHB/PEG(6%)/ALG-e(3%) film provided an increase in vapor permeation rate as compared to pure PHB film. Moreover, our films showed water vapor transmission rate values close to commercial skin dressings, e.g., 50 gm^−2^d^−1^ (hydrogel Bard) and 76 gm^−2^d^−1^ (hydrocolloid Dermiflex, Johnson & Johnson) [65]. This proves that, although the interstices or voids of the films’ structure can be occupied by AgNPs [66] hindering the flow of water vapor, our results remain acceptable in quality.

#### 3.3.5. Wettability

**Figure 7:**
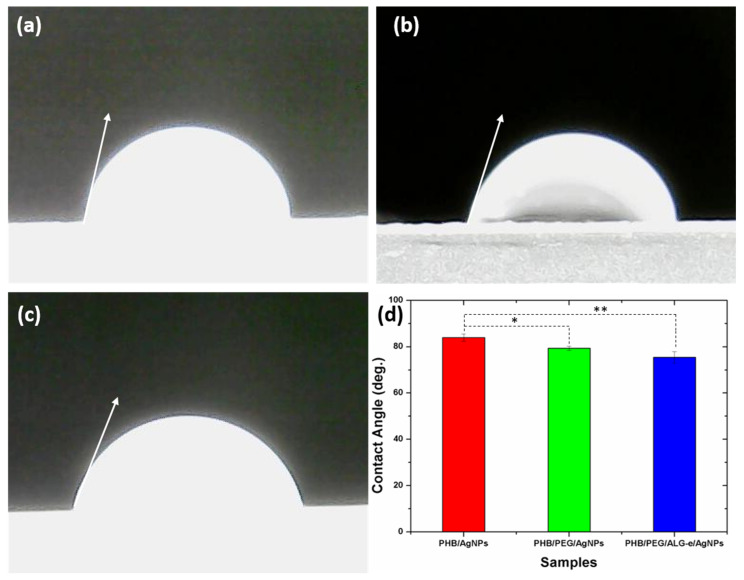
Representative contact angles of (**a**) PHB/AgNPs, (**b**) PHB/PEG/AgNPs, and (**c**) PHB/PEG/ALG-e/AgNPs and (**d**) their plotted average values. * and ** mean that there was statistical difference between the samples with significance level *p* < 0.05 or *p* < 0.01, respectively.

The wettability results of the films are presented in Figure 7. In Figure 7a–c, representative images of a droplet on the film surface are shown. The average contact angle value (N = 4) was estimated to be 84° ± 2° (PHB/AgNPs), 79° ± 1° (PHB/PEG/AgNPs), and 75° ± 2° (PHB/PEG/ALG-e/AgNPs). Although the values are in a close range, the ANOVA showed that there is a statistical difference between the samples. The contact angle value of the PHB/AgNPs film can be compared with values assigned to pure PHB, previously reported, e.g., 81° [8] and 82° [67]. 

It can be seen that the contact angle decreases significantly when PEG and ALG-e polymers are incorporated into the PHB structure. This shows that the wettability of the samples increases, that is, the films become more hydrophilic. This behavior is expected because PEG and ALG-e are known hydrophilic polymers, which provide a lower relative contact angle in the polymer blend [8]. The small difference observed between the values found here can be attributed to the small amounts of PEG and ALG-e used in the synthesis of the films. These results suggest that the PHB/PEG/ALG-e/AgNPs film is more hydrophilic, which matches with its better WVP properties.

### 3.4. Antimicrobial Properties

**Figure 8:**
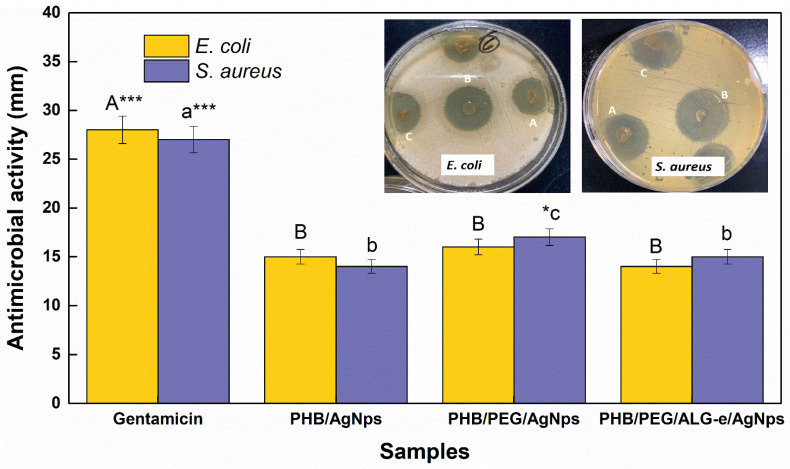
Antimicrobial activity of PHB-based blends loaded with AgNPs. The inserted images show one representative antimicrobial inhibition halo generated by A-PHB/AgNPs, B-PHB/PEG/AgNPs, and C-PHB/PEG/ALG-e/AgNPs against *E. coli* and *S. aureus* microorganisms. The statistically significant differences between the samples have been identified with different group letters. * and *** mean that there were significance levels *p* < 0.05 or *p* < 0.001, respectively.

The antimicrobial activity of the films was evaluated against some known strains using a convenient agar-diffusion method, whose results are shown in Figure 8. In this regard, all films produced a good antimicrobial effect on their surface against *E. coli* and *S. aureus* microorganisms, which can be ascribed to the dispersion of AgNPs distributed over their surface. This is because AgNPs modify the bacterial cell walls or prevent RNA replication, which further can lead to its death [68,69]. However, the different Ag contents loaded in the films were not sufficient to produce an inhibitory halo superior to gentamicin antibiotics. Inhibitory halos of 15.0 ± 0.8 mm (PHB/AgNPs), 16.0 ± 0.8 mm (PHB/PEG/AgNPs), and 14.0 ± 0.7 mm (PHB/PEG/ALG-e/AgNPs) were found against Gram negative *E. coli*, whereas against Gram positive *S. aureus* the values were found to be 14.0 ± 0.7 mm, 17.0 ± 0.9 mm, and 15.0 ± 0.8 mm, respectively. Thus, the best antimicrobial activity is assigned to the PHB/PEG/AgNPs sample, while PHB/AgNPs and PHB/PEG/ALG-e/AgNPs show similar behavior (*p* > 0.05). Although PHB/PEG/ALG-e/AgNPs film exhibited the best physicochemical properties, the SEM images reveal that this film has a surface structure similar to the PHB/AgNPs film (Figure 4). However, the PHB/PEG/AgNPs film exhibited a more porous structure, which may explain a greater interaction and release of AgNPs in the medium. Thus, the greater dispersion of AgNPs in the medium due to the more porous character of the PHB/PEG/AgNPs film leads to a greater number of bacterial deaths, which produces the greatest halo of antimicrobial inhibition of this film.

The inhibitory halo found for our PHB/AgNPs film is higher than the 1.5 mm value reported for the PHB/Ag nanocomposites fabricated recently by Jayakumar et al. [70]. Slepicka et al. [44] demonstrated that their PHB-based films loaded with AgNPs were effective against *E. coli* microorganisms, but with an inhibitory halo inferior to the control experiment, which is in agreement with our results. Chen et al. [71] proved that their chitosan films loaded with AgNPs killed 100% and 99% of *E. coli* and *S. aureus* microorganisms, respectively, suggesting that the incorporation of AgNPs plays a critical role for the antimicrobial activity of polymeric films. Furthermore, the chitosan-dialdehyde cellulose nanocrystal-silver nanoparticle films of Dong et al. [72] produced inhibition halos of 8.13 mm and 6.71 mm against *S. aureus* and *E. coli* microbes, respectively. Notably, such values are also lower than those reported in the present work, showing that the functionalization of our blends with AgNPs provides a robust antimicrobial effect. Comparing the physicochemical and antimicrobial properties of the films, we can conclude that although PHB/PEG/AgNPs showed the best antimicrobial performance, the PHB/PEG/ALG-e/AgNPs sample displayed the best physicochemical properties, suggesting that both samples arise as promising materials for application as a skin dressing.

## 4. Conclusions

We have successfully fabricated PHB/PEG/ALG-e blends loaded with AgNPs using an easy typical casting method. The UV-vis spectra of film-forming solutions revealed that AgNPs are loaded in different percentages into the blends. Reflections associated with an orthorhombic structure (space group P212121) of PHB were observed in the XRD patterns, which also had a superimposed peak at 38.1° ascribed to AgNPs. The PHB/PEG/ALG-e/AgNPs film has the least porous morphology of all samples, with pores having irregular shapes and different sizes. The chemical structure of the films displayed several vibrations related to the major polymer PHB, where an increase in the bands related to the amorphous and crystalline regions of the polymer was observed when PEG and ALG-e were incorporated. The thermal degradation of the films occurs due to random cleavage in the ester bond at temperatures above 200 °C, resulting in a similar thermal stability of the PHB films containing PEG and ALG-e polymers. The PHB/PEG/ALG-e/AgNPs film also showed the best water vapor permeability properties and high wettability, indicating greater hydrophilicity. All films exhibited antimicrobial activity against *S. aureus* and *E. coli*, as a result of the dispersion of AgNPs present in the surface of the films. Our findings suggest that PHB/PEG/ALG-e/AgNPs composite arises as a promising material for skin dressing purposes. Such results show that PHB/PEG/ALG-e blends loaded with Ag NPs acquired the ability to promote active surfaces with self-disinfecting properties, which can be useful for the development of new materials that can be applied in some relevant fields, e.g., tissue engineering, controlled drug delivery, and viral infection control. Therefore, our preliminary study suggests that the use of Ag NPs functionalizes the surface of PHB/PEG/ALG-e blends, promoting a robust antibacterial control.

## Figures and Tables

**Table 1:** Relationship between the amounts of the polymers used in the preparation of film-forming precursor gels.

Sample	PHB (%)	PEG (%)	ALG-e (%)
PHB/AgNPs	100%	-	-
PHB/PEG/AgNPs	90%	10%	-
PHB/PEG/ALG-e/AgNPs	93%	6%	1%

**Table 2:** Functional group assignment from FTIR Spectra of esterified (ALG-e) and pristine (ALG) alginate.

	Wavenumber (cm^−1^)			
Functional Group (Assigned Component)	ALG	ALG-e	Ref. [8]	Ref. [28]
Carboxylates (symmetrical stretching of COO^−^)	1614	1617	1612	1604
Carboxylates (asymmetric stretching of COO^−^)	1416	1400	1420	1413
Ether (stretching of C–O–C)	1033	1031	1025	1027
Guluronic and mannuronic acids residues	892, 944	890, 943	891, 943	940, 960
Ester (stretching of C=O)	-	1735	1736	1736
Ester (axial deformation of C–O)	-	1138	1134	1138

**Table 3:** Position of the IR bands found for the analyzed films.

	Wavenumber (cm^−1^)		
Functional Group (Assigned Component)	Present Work	Ref. [8]	Ref. [57]
Axial deformation of C=O ester group	1721	1721	1726
Axial deformations of C–O–C ester group	1278, 1129	1278–1130	-
Stretch of C–O bond	1055	1055	1050
Asymmetric angular deformations of –CH_3_ group	1458	1459	-
Symmetric angular deformations of –CH_3_ group	1378	1380	-
Axial deformation of C–C bond	979	978	-

**Table 4:** Comparative analysis of the thermal behavior of PHB, PHB/PEG, and PHB/PEG/ALG-e blends unloaded and loaded with Ag NPs.

	Temperature (°C)	
Samples	T_onset_	T_onset_ Ref. [8]
PHB	-	281
PHB/PEG	-	272
PHB/PEG/ALG-e	-	275
PHB/AgNPs	223	-
PHB/PEG/AgNPs	200	-
PHB/PEG/ALG-e/AgNPs	215	-

## Data Availability

Not applicable.
